# Mortality transition over a quarter century in rural South Africa: findings from population surveillance in Agincourt 1993-2018

**DOI:** 10.1080/16549716.2021.1990507

**Published:** 2022-04-04

**Authors:** Chodziwadziwa Whiteson Kabudula, Brian Houle, Daniel Ohene-Kwofie, Daniel Mahlangu, Nawi Ng, Hoang Van Minh, Francesc Xavier Gómez-Olivé, Stephen Tollman, Kathleen Kahn

**Affiliations:** a MRC/Wits Rural Public Health and Health Transitions Research Unit (Agincourt), School of Public Health, Faculty of Health Sciences, University of the Witwatersrand, Johannesburg, South Africa; b School of Demography, The Australian National University, Canberra, Australia; c CU Population Center, Institute of Behavioral Science, University of Colorado at Boulder, Boulder, Colorado, USA; d Department of Epidemiology and Global Health, Umeå University, Umeå, Sweden; e School of Public Health and Community Medicine, Institute of Medicine, Sahlgrenska Academy, University of Gothenburg, Gothenburg, Sweden; f Center for Population Health Sciences, Hanoi University of Public Health, Ha Noi, Vietnam

**Keywords:** South Africa, mortality, verbal autopsy, non- communicable diseases (NCDs), health and socio-demographic surveillance system (HDSS)

## Abstract

**Background:**

Mortality burden in South Africa since the mid-1990s has been characterized by a quadruple disease burden: HIV/AIDS and tuberculosis (TB); other communicable diseases (excluding HIV/AIDS and TB), maternal causes, perinatal conditions and nutritional deficiencies; non-communicable diseases (NCDs); and injuries. Causes from these broad groupings have persistently constituted the top 10 causes of death. However, proportions and rankings have varied over time, alongside overall mortality levels.

**Objective:**

To provide evidence on the contributions of age and cause-of-death to changes in mortality levels in a rural South African population over a quarter century (1993–2018).

**Methods:**

Using mortality and cause-of-death data from the Agincourt Health and Socio-Demographic Surveillance System (HDSS), we derive estimates of the distribution of deaths by cause, and hazards of death by age, sex, and time period, 1993–2018. We derive estimates of life expectancies at birth and years of life expectancy gained at age 15 if most common causes of death were deleted. We compare mortality indicators and cause-of-death trends from the Agincourt HDSS with South African national indicators generated from publicly available datasets.

**Results:**

Mortality and cause-of-death transition reveals that overall mortality levels have returned to pre-HIV epidemic levels. In recent years, the concentration of mortality has shifted towards older ages, and the mortality burden from cardiovascular diseases and other chronic NCDs are more prominent as people living with HIV/AIDS access ART and live longer. Changes in life expectancy at birth, distribution of deaths by age, and major cause-of-death categories in the Agincourt population follow a similar pattern to the South African population.

**Conclusion:**

The Agincourt HDSS provides critical information about general mortality, cause-of-death, and age patterns in rural South Africa. Realigning and strengthening the South African public health and healthcare systems is needed to concurrently cater for the prevention, control, and treatment of multiple disease conditions.

## Background

Ongoing characterization of mortality and disease patterns is required in different settings in order to set locally relevant health and development priorities, identify critical elements for strengthening of health systems, and determine the focus of health services and programmes. During the first half of the twentieth century, populations in high-income countries moved from periods of high, fluctuating mortality rates dominated by epidemics of infectious diseases, famines, and wars to periods of progressive reductions in mortality rates and the emergence of degenerative diseases as the major causes of morbidity and death [1]. They subsequently arrived at an era where mortality rates stabilized at relatively low levels and non-communicable diseases (NCDs) such as cardiovascular diseases, diabetes and cancers, and accidents became the main causes of death [1–3]. The transition in mortality levels and causes of death is still underway in low- and middle-income countries, with more varied progression compared to high-income countries. Changes in mortality and disease patterns in most low- and middle-income countries indicate that the transition is characterized by partial changes, reversals in the shift in the leading causes of death, and simultaneous occurrence of infectious and chronic diseases as the leading causes of morbidity and death [4–14].

**Figure 1.**
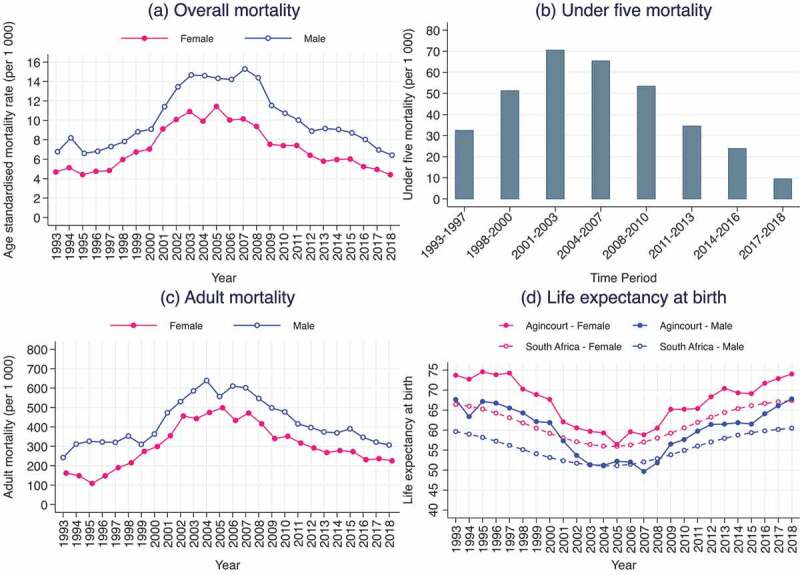
Trends in selected mortality indicators, Agincourt, South Africa, 1993–2018. (a) Overall mortality, (b) Under five mortality, (c) Adult mortality, and (d) Life expectancy at birth in Agincourt versus South Africa.

In South Africa, overall mortality levels were declining, and life expectancy was steadily improving from the 1960s until the early 1990s [15]. From the mid-1990s to the mid-2000s mortality levels steadily increased and life expectancy progressively declined – with the highest overall mortality and lowest life expectancy at birth levels around 2005–2007 as shown in Figure 1 [6,15–20]. At the national level, age-standardized death rates increased from 1 215 per 100 000 people in 1997 to a peak of 1 670 per 100 000 people in 2006, and life expectancy at birth declined from 63 years in 1995 to 54 years in 2005 [21,22]. Since then, overall mortality levels have been declining and life expectancy at birth steadily rising.

The dramatic increases in overall mortality and reductions in life expectancy from the mid-1990s to the mid-2000s were driven mostly by increases in mortality caused by the HIV/AIDS epidemic and lack of treatment programmes [16–18,23–25]. Although HIV/AIDS remained the single leading cause of death during the period 1997–2012, the overall mortality burden in South Africa since the mid-1990s has been characterized by a unique quadruple disease burden consisting of HIV/AIDS and tuberculosis (TB); other communicable diseases (excluding HIV/AIDS and TB), maternal causes, perinatal conditions and nutritional deficiencies; NCDs; and injuries. Causes from these four broad cause groupings persistently constituted the top 10 single causes of death over the period 1997–2012 [21]. However, their proportions and rankings varied over time alongside changes in overall mortality levels. The aim of this study is to provide evidence on the contributions of age and causes-of-death to changes in mortality levels in a rural South African population over a period of two and half decades, 1993–2018. This period spans socio-political change from apartheid to democracy (the country’s first democratic election was in 1994) and the emergence of the HIV/AIDS epidemic. We use life expectancy as a quantitative measure of mortality with age standardisation [26]. The study, based on rigorous and comprehensive health and socio-demographic surveillance data covering periods of different levels of the HIV epidemic and the availability of antiretroviral treatment, updates and extends published trends in mortality and cause-of-death profiles in the rural Agincourt population of Bushbuckridge sub-district, Mpumalanga Province, northeast South Africa.

## Methods

We used mortality and cause of death data collected from 1993 to 2018 as part of annual updates of vital events of the population of the Agincourt Health and Socio-Demographic Surveillance System (HDSS) in rural northeast South Africa [27,28]. Similar to earlier studies conducted in Agincourt [17,29–31], a person-year file was constructed containing one record for each year lived by each individual in the study population during the period 1993–2018. Attributes contained in each record consisted of Individual ID, sex, date of birth, date of death, age, calendar year, if the person died within the year, and the most probable cause-of-death.

The most probable cause-of-death was generated using the InterVA-5 probabilistic model (version 5.1) [32]. For each death, the InterVA-5 model assigns up to three likely causes of death with associated likelihoods based on information on signs and symptoms of the illness or injury prior to death collected through verbal autopsy (VA) interviews. The VA interviews were conducted with caregivers of individuals identified as having died between annual surveillance update rounds using a locally validated VA instrument until 2011 and WHO VA instruments from 2012 onwards [28,33]. The timing of the interviews ranged from 1 to 11 months after death. An indeterminate cause was assigned when the VA information was inadequate for the model to arrive at any cause of death. While causes of death derived by the InterVA model have been found to not substantially differ from those generated by physician coding [34,35], the InterVA model also offers the benefit of assigning causes of death in a standardized, automated manner that is much quicker and more consistent compared to physicians. This feature is particularly desirable for assessing changes over time and across settings.

Using the person-year file, we estimated the hazards of death by age, sex and time-period using logistic regression models [36–40]. Thereafter, we used the estimated hazards of death to construct standard life tables and cause-deleted life tables to, respectively, derive estimates of life expectancies at birth and to assess potential gains in life expectancy (PGLE) at age 15 if selected insignificant. Third, the InterVAcauses of death were eliminated. The PGLE provides a hypothetical estimate of the impact of a particular disease on life expectancy by highlighting the loss of life expectancy caused by a certain disease and provides a numerical indicator of survival if the disease is eliminated [41]. We follow methods that have been used in several other settings to assess the impact on life expectancy of various diseases, including cardiovascular diseases, neoplasms, HIV/AIDS and accidents using PGLE [41–44].

We split the calendar years into the following time periods: 1993–1997, 1998–2000, 2001–2003, 2004–2007, 2008–2010, 2011–2013, 2014–2016 and 2017–2018 to contextualize the dynamics of the HIV epidemic and the rollout of prevention of mother-to-child transmission (PMTCT) and antiretroviral treatment (ART) services. Where possible, we compared the indicators of mortality and cause-of-death trends from Agincourt with South African national indicators generated from publicly available datasets as a way of assessing the generalizability of our findings. We compared estimates of life expectancy at birth from the Agincourt HDSS with estimates of life expectancy at birth for South Africa obtained from the World Bank data archive [45]. We also compared the percentage distribution of deaths due to communicable diseases (Group I), non-communicable diseases (Group II) and external causes (Group III) by year of death from the Agincourt HDSS population with those in South Africa compiled by Statistics South Africa and archived in the DataFirst online microdata library [46]. Even though deaths due to HIV/AIDS and TB dominated by far deaths due to communicable diseases during periods of increased mortality [17], we did not separate HIV/AIDS and TB from communicable diseases in making the comparison due to known misattribution of HIV/AIDS deaths to infectious conditions, such as diarrhoea, tuberculosis and pneumonia in the South African national cause of death data [47–49]. Different time periods were used to compare the indicators of mortality and cause-of-death trends from Agincourt with South African national indicators due to the availability of publicly accessible national data.

## Software

All analyses have been conducted using Stata version 14.1 (Stata Corp., College Station, USA).

## Results

### Trends in overall mortality and life expectancy at birth

A total of 17 934 (8 660 in females and 9 274 in males) deaths were recorded in 2 210 631 (1 148 736 in females and 1 061 895 in males) person-years of follow-up in the Agincourt HDSS surveillance population over the period 1993–2018. Panel (a) of Figure 1, which shows year-by-year changes in age standardised mortality rates per 1 000 person-years, reveals steady increases in overall mortality from 1995 to 2005 for females and 2007 for males. For males, age standardised mortality rate increased from 6.6 deaths per 1 000 person-years in 1995 to 15.28 deaths per 1 000 person-years in 2007. For females, age standardised mortality rate increased from 4.4 deaths per 1 000 person-years in 1995 to 11.4 deaths per 1 000 person-years in 2005. Since peak levels, age standardised mortality rate has steadily declined in both males and females. As of 2018, age standardised mortality rate had declined to 5.8 and 5.7 deaths per 1 000 person-years for males and females, respectively.

Trends in mortality levels in children under the age of 5 years show progressive increases between 1993–1997 and 2001–2003 and decreases thereafter (Panel (b) of Figure 1). Notably, mortality in this age group more than doubled between 1993 and 2001 and decreased to a level lower than that in 1993 by 2017. As also shown in Panel (c) of Figure 1, for both males and females, adult (ages 15–64) mortality rates (per 1 000) also increased steadily from 1993 to 2004 and has been declining steadily since 2007. However, as of 2018, the level of adult mortality in both males and females was higher than it was in 1993.

Trends in life expectancy at birth exhibit a similar pattern to trends in overall mortality for both males and females (Panel (d) of Figure 1). For females, life expectancy at birth dropped from 74.8 years in 1995 to 56.4 years in 2005 (a loss of 18.4 years) and then steadily increased afterward to 74.0 years by 2018. For males, life expectancy at birth dropped from 67.1 years in 1995 to 49.7 years in 2007 (a loss of 17.4 years) and recovered to 67.7 years by 2018. Throughout the years, males persistently experienced significantly lower life expectancy at birth than females indicated by the non-overlapping confidence intervals between the lines for males and females, with differences ranging from 4.2 to 9.4 years.

Panel (d) of Figure 1 also compares trends in estimates of life expectancy at birth in the Agincourt HDSS population with estimates of life expectancy at birth in South Africa obtained from the World Bank data archive. Although for most of the years estimates of life expectancy at birth for the Agincourt HDSS population are higher than the estimates for South Africa, the changes over time follow a similar pattern. Both the Agincourt HDSS and South African series show steady reductions in life expectancy at birth from 1993 to 2005–2007 and steady increases thereafter. Both series also show that by 2018 life expectancy at birth had returned to the level it was in 1993.

## Distribution of deaths by cause categories

**Figure 2.**
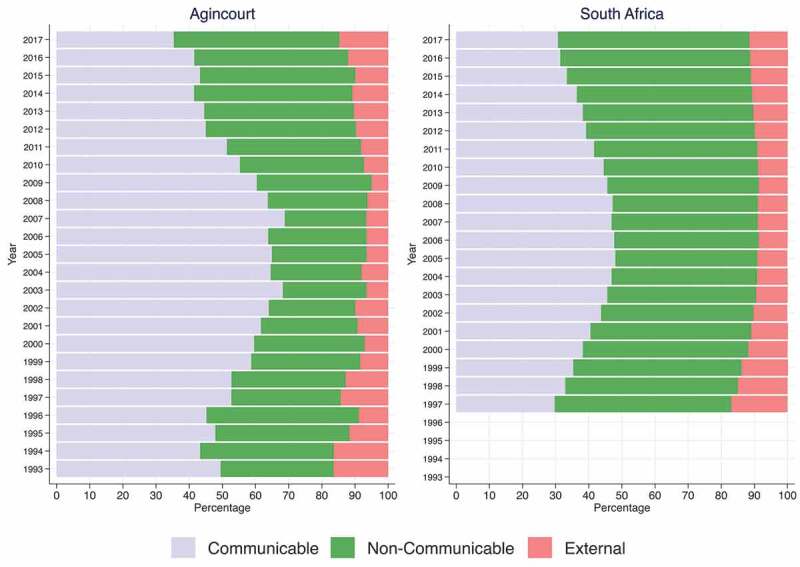
Distribution of deaths by cause categories in Agincourt versus South Africa 1993–2017.

Figure 2 shows the percentage distribution of deaths in three broad cause-of-death categories (communicable diseases (Group I), non-communicable diseases (Group II) and external causes (Group III)) by year of death in the Agincourt HDSS population. The percentage of deaths from communicable diseases steadily increased from 43% in 1994 to 69% by 2007 and steadily declined afterwards to 35% by 2017. The percentage of deaths from non-communicable diseases increased slightly from 34% in 1993 to 45% by 1996, progressively reduced to 25% by 2007 and steadily increased thereafter to 50% by 2017. The percentage of deaths from external causes progressively decreased from 16% in 1993 to 5% by 2009 and steadily increased thereafter to 15% by 2017. Figure 2 also shows that the overall changes over time in the percentage distribution of deaths due to the three broad cause categories in the Agincourt HDSS population follow a similar pattern to the changes in the South African population, although the percentages are different. For South Africa: (i) the percentage of deaths from communicable diseases increased from 29% in 1997 to 48% by 2005 and steadily declined afterwards to 31% by 2017; (ii) the percentage of deaths from non-communicable diseases progressively declined from 53% in 1997 to 42% by 2005 and steadily increased afterwards to 58% by 2017; and (iii) the percentage of deaths from external causes progressively decreased from 17% in 1997 to 8% by 2006 and steadily increased afterwards to 12% by 2017.

**Figure 5.**
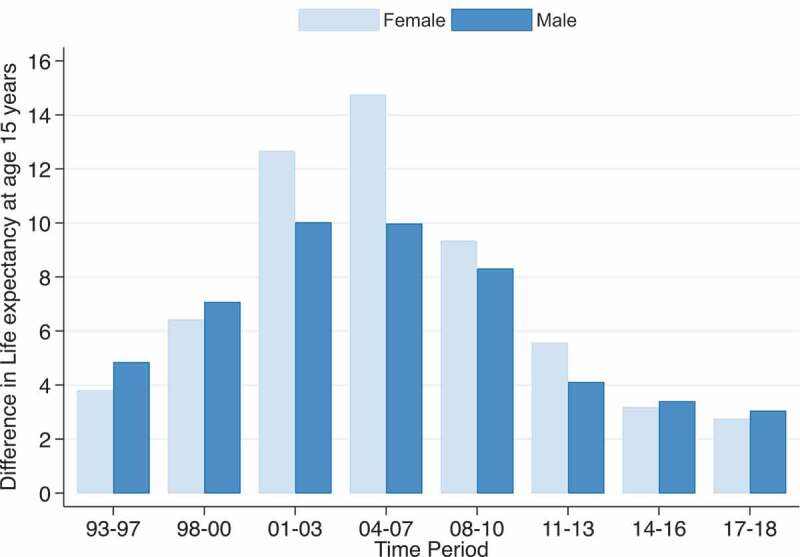
Potential increases in life expectancy at age 15 years that would result if deaths due to HIV/AIDS and TB were eliminated for males and females 1993–2018, Agincourt South Africa.

**Figure 3.**
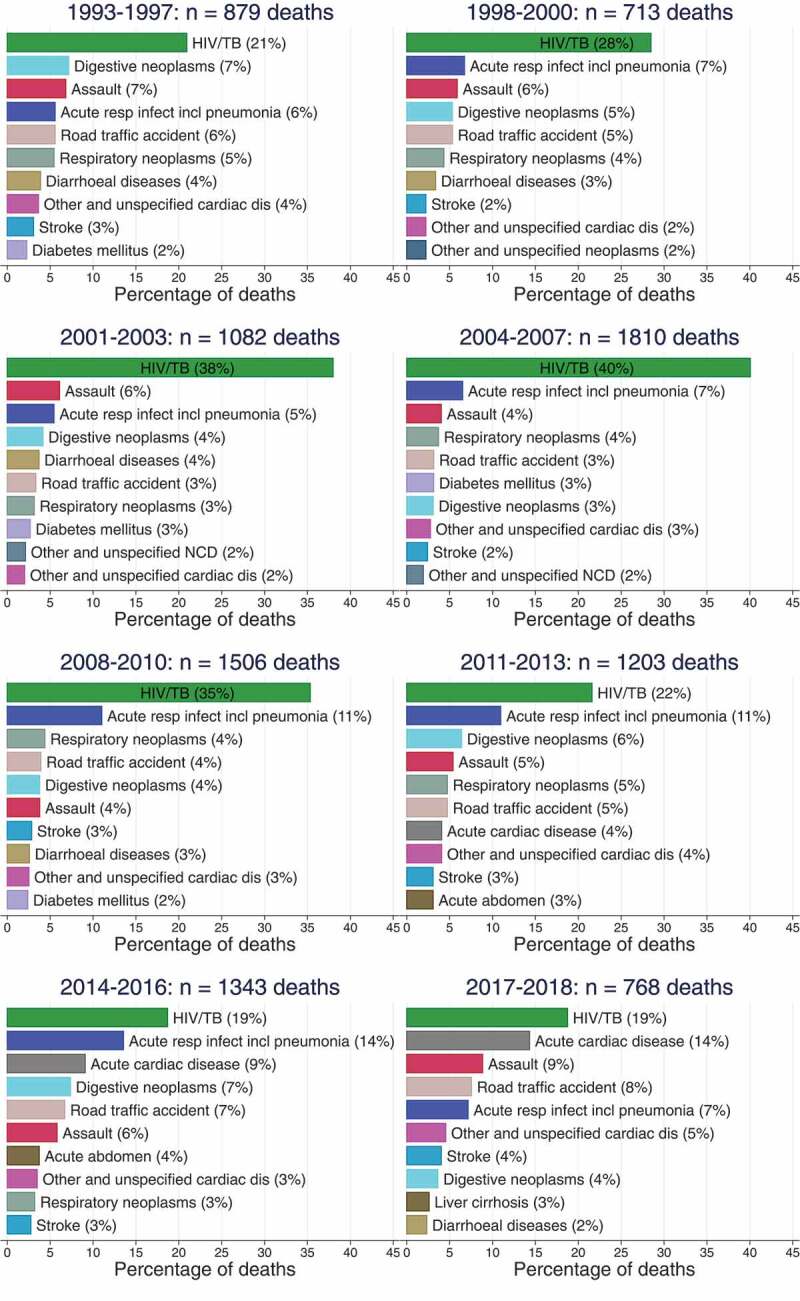
Leading causes of death in males 1993–2018, Agincourt South Africa.

**Figure 4.**
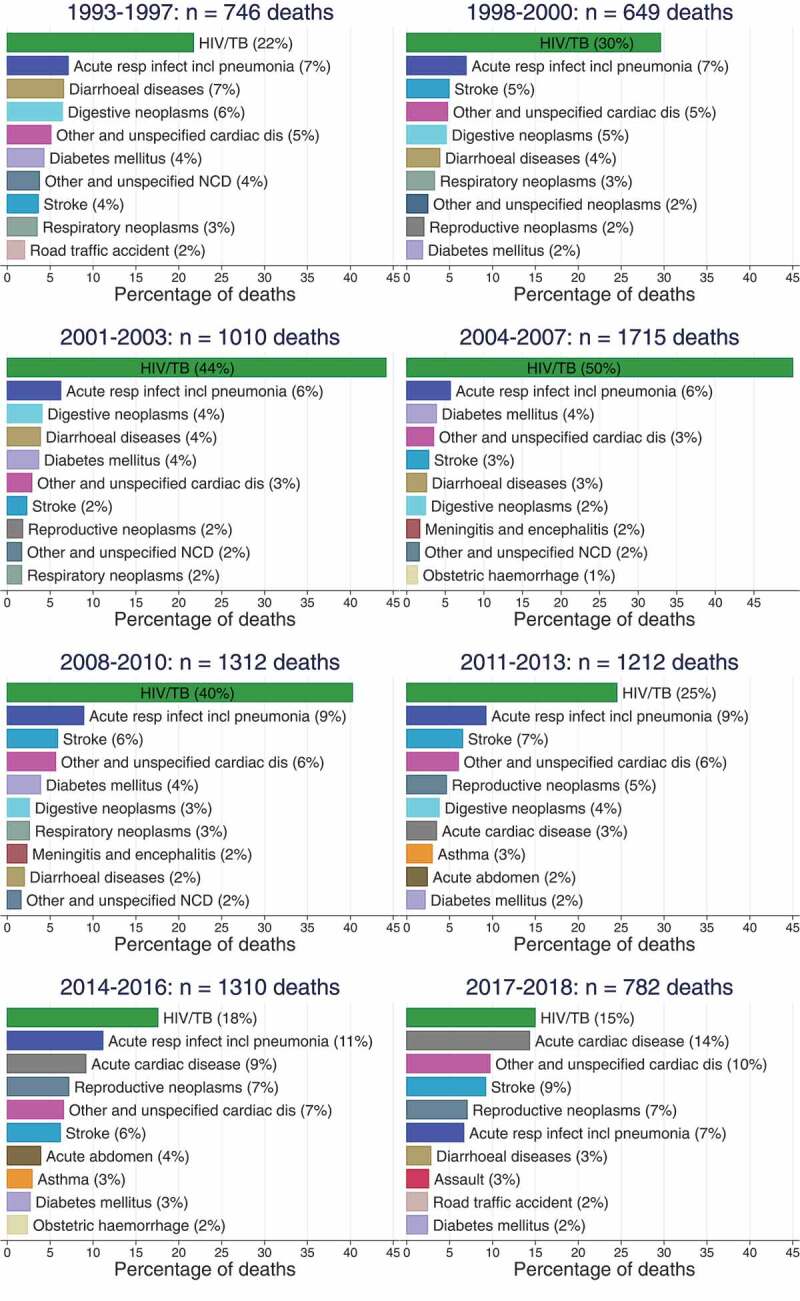
Leading causes of death in females 1993–2018, Agincourt South Africa.

Figures 3 and 4 present results on the evolution of the distribution of deaths by the top 10 leading single causes of death by sex and time period. The results presented in the figures exclude 12% of all deaths in the Agincourt HDSS population that either did not have verbal autopsies or were assigned an indeterminate cause of death by the InterVA model. Throughout all time periods, HIV/AIDS and TB feature as one of the top three single causes of death for both males and females. For males, the proportion of HIV/AIDS and TB deaths increased from 21% in 1993–1997 to 40% in 2004–2007, and began declining afterwards reaching 19% in 2017–2018. For females, the proportion of HIV/AIDS and TB deaths increased from 22% in 1993–1997 to 50% in 2004–2007, and progressively declined afterwards reaching 15% in 2017–2018. The results also show that while NCDs have been significant causes of death along with HIV/AIDS and TB over the 25-year period 1993–2018, cardiovascular diseases, such as stroke, and cardiac diseases have gained more prominence in recent years, especially among females. In 2017–2018, the proportion of cardiovascular disease deaths was at least 23% among males and 33% among females. In addition, the results also show the increased importance of road traffic accidents and assaults as leading causes of death among males. Combined, road traffic accidents and assaults contributed to 17% of the deaths in 2017–2018 among males compared to 5% of the deaths among females.

## Major cause of death-group contributions to life expectancy losses at age 15 years

Owing to the significant contribution of HIV/AIDS and TB to the mortality profiles of the Agincourt HDSS population in the last two and a half decades, Figure 5 presents the potential increases in life expectancy at age 15 years that would result if deaths due to HIV/AIDS and TB were eliminated. The results indicate that the elimination of HIV/AIDS and TB during the peak period of HIV/AIDS and TB mortality in 2004–2007 would have added 10 and 14.7 years to male and female life expectancies at age 15 years, respectively. Following the reduction in HIV/AIDS and TB mortality, in 2017–2018 the elimination of HIV/AIDS and TB would have added 3.0 years to male and 2.7 years to female life expectancy at age 15 years, which are lower than the 4.8 years for males and 3.8 years for females in 1993–1997.

**Figure 6.**
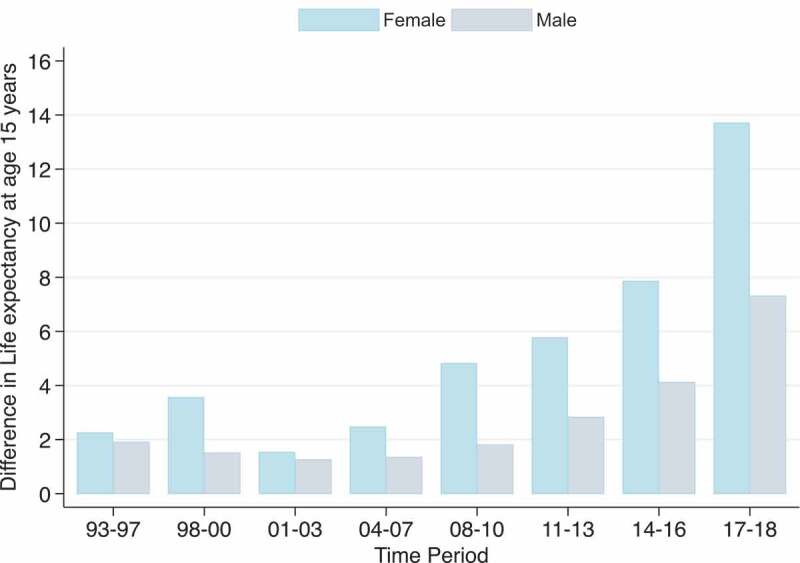
Potential increases in life expectancy at age 15 years that would result if deaths due to cardiovascular diseases were eliminated for males and females 1993–2018, Agincourt South Africa.

Building on the finding that cardiovascular diseases have progressively gained prominence among the leading causes of death, Figure 6 shows the potential increases in life expectancy at age 15 years that would result if deaths due to cardiovascular diseases (CVD) were eliminated. Even though beyond the scope of this paper, these results show what life expectancy would potentially be if the main risk factors for cardiovascular diseases, such as high blood pressure, diabetes, high cholesterol and smoking, were eliminated. The results show progressive increases in the impact of CVD on life expectancy at age 15 years from 2001 to 2018, with the highest impact among females. The years of potential increases in life expectancy at age 15 years that would result if deaths due to cardiovascular diseases were eliminated increased from 1.5 years in females and 1.3 years in males in 2001–2003 to 7.3 years in males and 13.7 years in females by 2017–2018.

## Discussion

A clear understanding of the levels and patterns of mortality and diseases in different sub-populations is continuously needed in order to formulate and implement locally relevant policies and programs to improve population health. However, effective responses to this need for populations in the most resource-poor settings are hampered by a lack of comprehensive, reliable, population-based data on health risks, exposures, and outcomes. One of the viable alternatives to addressing this lack of data has been the establishment of HDSSs in some resource-poor settings. HDSSs enumerate populations in geographically well-defined areas and prospectively collect detailed information on the core components of population change (births, deaths, and migrations) as well as complementary information on health, social and economic indicators [50–53]. In this study, we have used data from one of the longest-running HDSSs in sub-Saharan Africa, the Agincourt HDSS, to provide the latest evidence of the contributions of age and various causes of death to changes in mortality levels in a rural population in northeast South Africa from 1993 to 2018, a period of rapid health and social change. We have also compared the indicators of mortality and cause-of-death trends from the Agincourt HDSS with South African national indicators generated from publicly available datasets as a way of assessing the generalizability of our findings. The period covered in this study includes the start of the HIV/AIDS epidemic, its rapid spread and evolution, delayed introduction and eventual widespread rollout of ART, coupled with broader demographic, socioeconomic, technological, political, and cultural changes.

Unlike prior research [6,17,25,30,54,55], our study more fully captures the effects on mortality levels and causes-of-death during the public sector rollout of ART in Agincourt. The findings in this paper reveal that overall mortality levels have returned to pre-HIV epidemic levels; the concentration of mortality is shifting towards older age groups and the mortality burden from CVD and other chronic NCDs are becoming more prominent as larger numbers of people living with HIV/AIDS access ART and live longer. The increased prominence of CVD and other chronic NCDs as leading causes of death emerging in Agincourt was noted much earlier in communities in Southeast Asia where the progression of the transition in mortality levels and causes of death is much advanced [56].

While there have been impressive reductions in mortality levels in Agincourt since 2007, the COVID-19 pandemic that has been underway in South Africa since March 2020 may well induce some reversal through both direct and indirect mortality, the latter resulting from reduced provision and utilization of health service for NCDs, HIV/AIDS, TB, antenatal care and vaccine-preventable diseases in children under 5 years [57]. The extent of such reversal for different age and sex groups will be captured by the HDSS owing to its versatility and longitudinal, continuous nature.

A previous study showed broad similarity in major cause of death categories despite low agreement of cause attribution at the individual level between the causes of death in the South African national data that are determined from death notification forms (Form BI-1663) completed by clinicians or death reports (Form BI-1680) completed by authorised traditional leaders when clinicians are not available, and causes of death in the Agincourt HDSS population determined from verbal autopsies using the InterVA model [49]. The striking similarities in the general pattern of changes in life expectancy at birth and major cause of death categories between the Agincourt HDSS population and the South African population in the present study further illustrate the utility of the Agincourt HDSS. The recent formation of a network of South African HDSSs and harmonization of their data collection protocols under the umbrella of the South African Population Research Infrastructure Network (SAPRIN) will furthermore strengthen the valuable contribution of HDSSs in understanding mortality, cause-of-death, and age patterns in South Africa. Although there may be a number of similarities between the Agincourt HDSS area and other rural settings in South Africa in mortality and other health outcomes, the data from the SAPRIN HDSS nodes will illuminate important differences in various settings that may be masked by aggregated national data and could facilitate the formulation and implementation of better and locally relevant public health policies.

This study has a number of limitations. First, the sensitivity of our findings to different computer-based models for assigning causes of death other than the InterVA model, such as Tariff [58] and InSilicoVA [59], is not known. However, we do not expect the use of a different model to significantly alter our findings as the causes-of-death derived from the InterVA model were found to be not substantially different from those generated by physician coding in Agincourt and other settings [34,35]. A comparison of causes-of-death from the InterVA and InSilicoVA models also did not show substantial differences [60]. Second, there is the potential incomparability of causes-of-death derived by the InterVA model from the verbal autopsy data that were collected in the Agincourt HDSS using a locally validated VA instrument from 1993 to 2011 and WHO VA instruments from 2012 onwards [28,33]. The probability of the InterVA model to derive a specific cause-of-death using data from the later years that were collected using WHO VA instruments that have more questions relevant to the model might be different from that using data collected using the earlier locally validated VA instrument. However, the smooth transition in the changes in the leading causes of death over time provides assurance that the extent of such incompatibility is insignificant. Third, the InterVA model categorises neoplasms into those affecting the digestive, respiratory, breast, reproductive and other and unspecified sites. Previous work by Byass and colleagues [61,62–64] has shown some differences, though not substantial, in the cause-specific mortality fractions for various neoplasm categories generated by the InterVA model compared to those generated by physician coding. This suggests that the InterVA model may not sufficiently distinguish between cancer sites. Therefore, it is possible that the magnitude of some of the neoplasm categories in the top 10 leading single causes of death reported in this paper may be overestimated and some underestimated. However, given the consistency of the InterVA model in assigning causes of death over time, this would not affect the relative magnitude of the changes in the contribution of different categories of neoplasms to the overall mortality burden. Fourth, updates of vital events in the Agincourt HDSS occurred once a year. As a result, some stillbirths, neonatal and infant deaths may not have been recorded particularly when birth and death occurred between consecutive household visits [27]. This may explain why life expectancy in the Agincourt HDSS population appears to be unexpectedly high for a poor rural setting. In order to minimize this bias, since 2000 names of the most recent child born to each woman were added to the pre-populated household roster and since 2006 there has been careful probing for pregnancies and births since the last recorded child by asking about pregnancy status of every woman of childbearing age [27]. Finally, as previously stated [27], the use of proxy respondents when updating the household roster and vital events (common to all HDSS data collection methods) may reduce the accuracy of some individual-level information (e.g. dates of birth, migration and death).

## Conclusion

Our findings show that the population in Agincourt, a rural area of South Africa adjacent to southern Mozambique, has experienced significant shifts in mortality levels and the associated contributions of age and various causes of death over a quarter century of tremendous health, population and social change. The findings also illustrate that the general mortality and cause of death transition experienced in the Agincourt area largely reflects the mortality, age, and cause of death transition experienced in South Africa, although the magnitudes may differ from one setting to the other. Most importantly, the mortality and cause-of-death transition presented in this paper highlights the increased importance of CVD and other chronic NCDs as leading causes of death in recent years as more people living with HIV/AIDS access ART and attain prolonged survival. Therefore, there is a need for realigning and strengthening of the public health and healthcare systems in South Africa to concurrently cater for the prevention, control, and treatment of multiple disease conditions.

## Data Availability

Detailed documentation describing the Agincourt HDSS data and an anonymized database containing data from 10% of the surveillance households are available for public access on the MRC/Wits Rural Public Health and Health Transitions Research Unit (Agincourt)’s data repository [62]. The Agincourt HDSS core demographic data are also routinely deposited for public access in the INDEPTH Network Data Repository [63] and SAPRIN data repository [64]. The customized data used in this study will be made available on request to interested researchers.

## Paper context

South Africa is experiencing a protracted transition in mortality levels and causes of death, with multiple stages overlapping and changes incomplete. This requires continuous monitoring in order to advise policy-makers on health planning and resource allocation. Using health and socio-demographic surveillance data, this paper provides evidence on the contributions of age and various causes of death to changes in mortality levels in a rural population in northeast South Africa over a quarter century, 1993–2018.
